# Knowledge and behaviour as determinants of anti-malarial drug use in a peri-urban population from malaria holoendemic region of western Kenya

**DOI:** 10.1186/1475-2875-10-99

**Published:** 2011-04-20

**Authors:** Carren A Watsierah, Walter GZO Jura, Evans Raballah, Dan Kaseje, Benard Abong'o, Collins Ouma

**Affiliations:** 1 Great Lakes University of Kisumu, P.O. Box 2224-40100, Kisumu, Kenya; 2 Department of Zoology, Maseno University, Private Bag, Maseno, Kenya; 3 Department of Biochemistry and Biotechnology, Kenyatta University, P.O. Box 43844-00100 GPO, Nairobi, Kenya; 4 Department of Biomedical Sciences and Technology, Maseno University, Private Bag, Maseno, Kenya; 5 University of New Mexico/Centre for Global Health Research, Kenya Medical Research Institute, P.O. Box 1578-40100, Kisumu, Kenya

## Abstract

**Background:**

The appropriate use of anti-malarial drugs determines therapeutic efficacy and the emergence and spread of drug-resistant malaria. Strategies for improving drug compliance require accurate information about current practices at the consumer level. This is to ascertain that the currently applied new combination therapy to malaria treatment will achieve sustained cure rates and protection against parasite resistance. Therefore, this cross-sectional study was designed to determine knowledge and behaviour of the consumers in households (n = 397) in peri-urban location in a malaria holoendemic region of western Kenya.

**Methods:**

The knowledge and behaviour associated with anti-malarial use were evaluated. Using clusters, a questionnaire was administered to a particular household member who had the most recent malaria episode (within <2 weeks) and used an anti-malarial for cure. Mothers/caretakers provided information for children aged <13 years.

**Results:**

Consumers' knowledge on dosage and duration/frequency demonstrated that only 29.4% used the correct artemisinin-based combination therapy (ACT) dosage. Most respondents who used quinine identified the correct duration of use (96.4%) since its administration was entirely at health facilities. To assess behaviours during use of anti-malarial drugs, respondents were stratified into those who took drugs with prescription (39.4%) and without prescription (61.6%). For those without prescription, the reasons given were; procedure of acquisition less costly (39.0%), took same drug for similar symptoms (23.0%), not satisfied with health services (15.5%), neighbour/friend/relative previously taken the same drug (12.5%) and health institution was far from their location (10%).

**Conclusion:**

Majority of consumers in the study area were knowledgeable on the symptoms of malaria. In addition, majority acquired ineffective anti-malarial drugs for treatment and reported sub-optimal treatment regimens with the currently recommended drugs. Furthermore, behaviours which constrain the successful up-scaling of ACT were common, creating a challenge in the desire to turn efficacy to effectiveness of the combination therapy programme. It will be important to direct and focus interventions in creating awareness on the importance of using recommended drugs to lessen the use of less efficacious anti-malarials. In addition, the consumers need to be educated on the importance of drug adherence in such areas to reduce the emergence and spread of drug-resistant malaria.

## Background

The burden of malaria persists in many parts of Africa despite the availability of many interventions that are focused on preventive and therapeutic strategies [1]. For instance, the Roll-Back Malaria (RBM) initiative is working to improve prevention efforts in affected countries, through insecticide-treated nets (ITNs), indoor residual spraying (IRS) of pesticides, and intermittent preventive treatment (IPT) for pregnant women [1]. Other interventions focus on effective anti-malarial regimens like artemisinin-based combination therapy (ACT) and improving home management of the disease [2]. The development of resistance by malaria parasites to more affordable anti-malarial drugs such as chloroquine and sulphadoxine-pyrimethamine has called for the use of ACT as the most effective treatment option in sub-Saharan Africa [3]. Since most individuals in sub-Saharan Africa opt to treat malaria without visiting a medical facility, appropriate use of anti-malarial drugs is vital to effective treatment of the disease [2]. The correct use of anti-malarial drugs is not only key, not only to therapeutic success, but is also paramount in deterring the spread of drug-resistant malaria [4].

The pattern of anti-malarial drug use in endemic areas of Africa is quite different from the use of other drugs due to the high intensity of malaria transmission in these regions. In areas of intense malaria transmission, anti-malarial drugs are given repeatedly to treat frequent fevers (even in the absence of malaria) [5]. The practice of self-medication is also increasingly becoming a major health concern. Research findings show that many illnesses including malaria, are treated without consultations with health professionals [6]. In areas where malaria transmission is endemic, about 50-80% of people first visit private drug outlets for malaria treatment and use these anti-malarial drugs even without prescription [7]. Most studies in Kenya have examined retail traders and health care providers of anti-malarial drugs, in order to address the anti-malarial dispensing level [8-10]. However, consumers also play a critical role in determining how these drugs are used. Understanding the knowledge and behaviour of consumers and how they actually use antimalarial will aid in devising strategies to increase the correct use of these drugs.

The current study site, a peri-urban location within Kisumu, is situated in a *Plasmodium falciparum *holoendemic transmission area of western Kenya. Malaria in this region accounts for about 40% of out-patient visits, and about 40% of hospital in-patient admissions [11]. Malaria transmission occurs all year round, peaking in the rainy season months of April and May [12]. The National Malaria Control Programme in Kenya recommends ACT- artemether/lumefantrine as the first-line drug for uncomplicated malaria and for chemoprophylaxis in pregnancy. Quinine is the second line anti-malarial, and is the recommended treatment for severe and complicated malaria.

Studies have previously observed that anti-malarial drugs are bought from commercial pharmacists, street vendors, retail shops (e.g. grocery) or from licensed and unlicensed drug sellers [13,14]. Additional studies also observed that most patients reporting at the health facilities would have at least gone through home-based treatment or local drug shops initially [13,14]. Hence, there is concern that household and communities' patterns of use of anti-malarial drugs are often inadequate and inappropriate depending on the knowledge and behaviour adopted for use of other medicines, not only for malaria [13]. It is upon this background that the current study on behaviours and knowledge determining the patterns of anti-malarial drug use among the residents of a peri-urban region of Kisumu in western Kenya, was carried out.

## Methods

### Study setting

The current study was carried out in Manyatta-B sub-location in Winam division of Kisumu as previously described in our recent publication [14]. In brief, the area consists of three villages, Lower Kanyakwar, Upper Kanyakwar and Kuoyo, which are further divided into nine smaller units depending on the sub-clan boundaries. Within the sub-clan boundaries are informal dwellings. According to the last population census, the total number of inhabitants in this sub-location is 21,027 in 6,035 households [15]. The total area is 3.3 km^2 ^with a density of 6, 372 people/km^2 ^[15]. Housings consist, mostly, of rented rooms within an area of 10-20 m^2 ^for 4-8 persons in a household [14]. These individuals live in a congested and miserable conditions, with considerable lack of the basic necessities of life, such as clean piped-water and proper housing conditions [16]. There is poor drainage and lots of stagnant water, which form good breeding grounds all year round for malaria vectors [17].

### Study participants

In order to establish the knowledge and behaviour determining use of anti-malarial drugs in the study population at a point in time, residents living within households (n = 397) in the above described peri-urban region of Kisumu in western Kenya, were recruited. This was a descriptive cross-sectional survey in which sampling was carried out using cluster method as previously described [13,18]. A total of 6,035 households in nine clusters were sampled in order to achieve the required sample size as previously described [14]. Data were collected through interviews, using structured questions in selected households in which diagnosed or self-reported episodes of malaria occurred within the last two weeks prior to the interview dates.

Included in the study were individuals living in the above-described households and who had experienced malaria and used anti-malarial or other drugs perceived by them as anti-malarial, due to self/presumptive or laboratory diagnosis within the previous two weeks. In the absence of laboratory diagnosis, malaria according to residents in the study area was defined by either the presence or mixed symptoms of headache, high temperature, shivering, diarrhoea, vomiting and pain in the joints. However, if more than one individual in a household had experienced malaria-related symptoms and had used anti-malarial or other drugs viewed or perceived by them as anti-malarial within the described period (i.e. two weeks), then focus was on the most current episode, while the rest were excluded. This strategy was adopted so as to capture the most recent knowledge and behaviour associated with anti-malarial drug use and to minimize errors associated with recall. If no one had experienced malaria episodes within the previous two weeks, the interviewer moved to the next household in the cluster. Correct drug use was assessed by questionnaires through identification of the drug used and whether or not the respondents used it as per the prescription. The study was approved by the ethical and scientific review committees at the Kenya Medical Research Institute and the Great Lakes University of Kisumu Ethical Review Board (GERB), Kenya.

### Sampling design

As previously defined in our publication [14], the study employed cluster sampling method in selecting the households in the study area. A total of nine clan regions were chosen as clusters. The study units comprised households within the cluster that had its member experiencing malaria in the last two weeks. The probability of selection of the primary sampling units for each cluster was proportional to the estimated sample size. From each cluster, households were then selected on continuous basis until the total number (n = 397) required was achieved.

### Research procedure

The quantitative data, which were collected from 397 households, were obtained through interviews using structured questions. The tool for data collection was a pre-prepared questionnaire, which was administered to the particular household member, who had previous malaria episode and used anti-malarial or drug perceived to be an anti-malarial. However, in case of children below the age of 13 years, mothers or caretakers provided the information. Using clusters, households which had experienced malaria episodes were established first through identification interview, which involved all the households starting with exactly the first household visited in the cluster. The interview was then repeated in all the households identified until the required number was achieved. As stated above, particular emphasis was on the most recent episode of self/presumptive or laboratory diagnosed malaria in the household. Research/field assistants (n = 5) who were involved in data collection, used drug charts containing samples of commonly used anti-malarial drugs to aid in recall and to validate reports. In addition, remaining packages or tablets were reviewed where possible.

Prior to the actual study, the field assistants who were themselves residents of the study sub-location and with a minimum of form four level of education, were interviewed and recruited as enumerators. They were required to be fluent in English, Swahili and Dho-Luo languages. Training of the research assistants on survey interviewing techniques was carried out for one day followed by a second day of pre-testing of survey tools and methodologies in a neighbouring community with the same characteristics. Based on the experiences and results of the pre-test, further re-training and refining of techniques of interviewing and modification of research tools were performed.

### Statistical analyses

Statistical analyses were performed using SPSS (Version 15.0). Logistic regression analysis between the independent and dependent variables was used to identify variables associated with pattern of use of anti-malarial drugs. The variables included in the logistic regression model comprised knowledge of (symptoms for use, dosage, duration, anti-malarials availability apart from what was taken). The variables that were significantly associated with pattern of use (correct drugs taken, correct duration, and correct dosage) were further analyzed using Chi-square tests. Proportions on knowledge, drug-taking behaviour and correctness of patterns of use were determined. A *P*≤0.05 was considered statistically significant.

## Results

### The use of anti-malarial drugs

**Figure 1:**
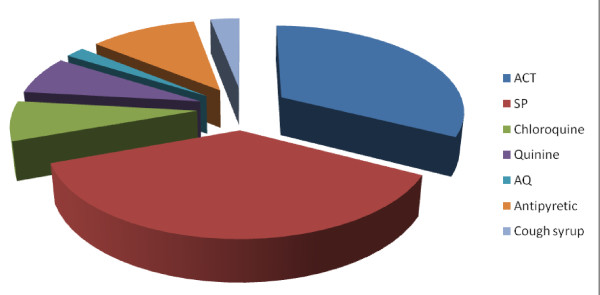
**Anti-malarial drugs used in households in Manyatta-B**. ACT-Artemether combined therapy, SP-Sulphadoxine-pyrimethamine, AQ-Amodiaquine. SP was the most commonly used anti-malarial drug, followed with ACT.

Overall, 32.5% of households in Manyatta-B acquired and used ACT as first choice of anti-malarial drug while 37.0% acquired and used SP. The use of ACT was reported mostly by users who acquired anti-malarials from government health institutions (42.1%) and from pharmacies/chemists (32.0%). Eighty three percent (83%) of the SP users cited low price of the drug and/or its availability at the source as the basis for the use of the drug. In general, the proportions of those who acquired other anti-malarials other than ACT and SP were comparable (chloroquine = 7.3%, quinine = 7.1%, AQ = 2.0%, and antipyretic = 11.1%) with 3.0% using cough syrup (perceived to be an anti-malarial) (Figure 1).

### Knowledge level and pattern of anti-malarial drugs use in Manyatta-B

In order to assess the knowledge level on drug used, the pattern of anti-malarial use (symptoms for use, dosage, duration/frequency and available anti-malarials) was evaluated. Table 1 presents data on the pattern of anti-malarial drugs used in Manyatta-B. Results revealed that most of the respondents who had taken anti-malarial drugs reported high temperatures (48.4%), headache (39.4%), vomiting (7.6%) and shivering (4.0%) as symptoms for anti-malarial drug use, while the rest (0.6%) reported other symptoms (coughs, pregnancy-related and acute respiratory infections).

**Table 1:** Anti-malarial drugs taken and the doses and duration with which they were used in Manyatta B.

Drugs taken	Duration of use		Dosage of use	
	
	Correct	Incorrect	Correct	Incorrect
**ACT**	42 (33.0%)	85 (67.0%)	37 (29.4%)	90 (70.6%)

**SP**	120(82.0%)	27 (18.0%)	125 (85.0%)	22 (15.0%)

**Quinine**	27 (96.4%)	1 (3.6%)	*	*

**Chloroquine**	10 (34.5%)	19 (65.5%)	15 (51.7%)	14 (48.3%)

**Amodiaquine**	*	*	*	*

ACT-Artemether combined therapy, SP-Sulphadoxine-pyrimethamine. Asterisks (*) represent no response or "I don't know". More users of quinine and SP could identify the correct duration of use.

Consumers' knowledge on the dosage and duration/frequency was further assessed on respondents who reported using the anti-malarial drugs. Most of SP consumers used the correct dosage (85.0%) and within the correct duration (82.0%) (Table 1). Only 29.4% of the consumers who reported having used ACT could identify the correct daily adult dose of 8 tablets (ACT-artemether/lumefantrine, commonly available in 20/120-mg tablet size) (Table 1). All other ACT users (70.6%) reported taking fewer than eight tablets a day. Similarly, of those who responded to the question of duration of ACT therapy, the majority (67.0%) responded incorrectly, mostly identifying shorter treatment duration (less than 3 days) (Table 1). Most respondents who used quinine could identify the correct duration of use (96.4%) since its administration was entirely done in health facilities (Table 1). All of the consumers that used amodiaquine either could not remember or declined to respond to questions on dosage and duration (Table 1).

### Behaviours in anti-malarial drug use

In this study, behaviour was defined as habitual practices, actions or deeds an individual or households carry out with an anti-malarial drug. In order to assess behaviours during the use of anti-malarial drugs, respondents were first stratified into those who took drugs with prescription (39.4%) and without prescription (61.6%). For those who had no prescription, the reasons given were as follows; 39.0% said procedure of acquisition was less costly, 23.0% took same drug for similar symptoms, 15.5% were not satisfied with health services/institutions, 12.5% had neighbour/friend/relative previously taking the same drug and 10% said health institution was far from their location.

In addition, there were notable differences between proportions of those who bought full dosage of drugs (39.1%) versus those who did not (60.9%). For those who did not buy full drug dosage, they gave the following reasons; 67.3% could not afford, 16.8% got better after taking few tablets, 14.4% said symptoms were mild and so there was no need of buying full dosage, while 1.4% gave other reasons not listed in the questionnaire. When asked about how drugs were taken, 63.9% reported to have taken drugs according to advice given at the source while 36.1% reportedly did not take drugs according to advice given. Reasons for not taking drugs according to advice were mainly; 41.4% kept drugs for future episodes of the same illness, 33.0% got better hence discontinued, and 16.2% shared the dosage with another person while the rest gave other actions not listed on the questionnaire.

When asked about the drug used, 34.6% of the respondents reported the drugs used for treatment was always available at source, 20.9% said the drug of has a faster action, 18.8% reported that they have been using the same drug for the same ailment/symptoms, 12.0% reported that the drug used had no side effects, 9.4% reported that the drug had fewer number of dosages per treatment course and 4.3% included other reasons. In terms of day of first use of drugs after the symptoms started, 34.3% took drugs the same day (within 12 hours of illness), 38.5% took drugs the next day (within 24 hours of illness), 17.6% took drugs two days after (within 48 hours of illness), 9.1% took drugs three or more days (60 hours or more), while 0.5% could not remember.

Finally, when asked about what the respondents would do if they missed their morning dose during treatment course, 32.7% said they would ignore their missed dose, 25.9% indicated they would take it immediately they remember, 21.2% said they would double the next dose, 15.6% said they would extend day of therapy, 2.8% said they would narrow the dosing interval, while 1.8% gave other actions.

### Association between knowledge factors and anti-malarial drug use patterns

In order to determine knowledge factors associated with patterns of use of anti-malarial drugs in this peri-urban population, a logistic regression analysis was performed. The independent variables included knowledge on symptoms for use, knowledge on dosage, knowledge on duration, and knowledge on available anti-malarials. The above factors were regressed against the outcome variables (i.e. drugs taken, duration of use and dosage/frequency of use) (Table 2).

**Table 2:** Knowledge factors and the use of anti-malarial drugs in Manyatta-B

	Dependent variables		
	
	Drugs taken	Dosage used	Duration used
**Constants**	2.021	1.231	1.778

**Knowledge variables**			

Symptoms for use	**β = 0.063, *P *= 0.001**	β = 0.016, *P *= 0.849	β = 0.079, *P *= 0.150

Anti-malarial(s) availability	**β = 0.013, *P *= 0.020**	**β = 0.121, *P *= 0.027**	β = 0.018, *P *= 0.722

Dosage	β = 0.101, *P *= 0.341	β = 0.115, *P *= 0.279	β = 0.105, *P *= 0.322

Duration	β = 0.192, *P *= 0.089	β = 0.039, *P *= 0.706	**β = 0.018, *P *= 0.022**

Logistic regression analysis between the independent and dependant variables was used to identify variables associated with the use of anti-malarial drugs, *P*-values in bold were statistically significant at *P *≤ 0.05. β = standard co-efficient. The result above shows that knowledge on symptoms for use and knowledge on anti-malaria available work together to influence types of anti-malarial that are taken in the households. Furthermore, knowledge on the duration of use determined the duration of which drugs were taken, while, knowledge on available anti-malarials determined the dosage used.

Knowledge on symptoms of malaria was a significant factor in determining the type of anti-malarial drug taken since many respondents took ACT (94.5%) and SP (93.2%) against the presence or mixed symptoms of headache, high temperature, shivering and vomiting as compared to other symptoms mentioned (β = 0.063, *P *= 0.001) (Table 2).

Furthermore, knowledge on the anti-malarial drugs available in the market significantly influenced the drug taken (β = 0.013, *P *= 0.020) and dosages used (β = 0.121, *P *= 0.027) (Table 2), primarily due to the fact that significantly higher proportions of respondents with knowledge on availability of SP (78.5%) took SP against symptoms of malaria and further took correct dosages as opposed to individuals with knowledge on other drugs (*P *= 0.002). Further analysis revealed that knowledge on duration of use significantly influenced the duration for which drugs were taken (β = 0.018, *P *= 0.022) since many individuals who used ACT took it for shorter (incorrect) duration as compared to the individuals who used other anti-malarials (Table 2).

## Discussion

This study has shown that populations living in malarial-prone areas are generally knowledgeable about the signs and symptoms of malaria such as high temperatures, headache and vomiting. These findings are consistent with other studies carried out in malaria endemic areas in which knowledge on symptoms of malaria was demonstrated [13,19,20]. For example, a study investigating the prevalence of malaria among clients seeking treatment for fever and/or malaria at drug store in rural Tanzania [20] demonstrated that 24.0% of the people who experienced fever and reported to drug stores to purchase anti-malarial drugs were actually diagnosed with malaria, even though 10.7% of them had reported before that they had malaria/fever [20].

The households which accessed anti-malarials with or without prescription in the current study were equally knowledgeable about the symptoms of malaria and would seek health care within 24 hours of onset of symptoms. While health seeking behaviour within the first 24 hours after the onset of symptoms is highly commendable for this community, the actions reported after missing a dose during the treatment course like ignoring a missed dose, doubling the next dose and narrowing a dosing interval are worrying and potentially requires an urgent public health intervention, which should include educating the local populations on the importance of taking drugs as prescribed. Furthermore, such informal use of anti-malarial could increase the risk of incorrect dosing, inappropriate treatment and interactions of different medicines, which could have a negative impact on malaria treatment safety and efficacy.

The findings in the current study further reveal that behaviour may influence the patterns of anti-malarial drug use. For instance, obtaining the anti-malarial drugs without prescription is more of a common practice than seeking prescription. This is not surprising given that other studies had consistently demonstrated that self-medication is common in most malaria-prone regions [13]. Consistent with our observations, other studies carried out in malaria endemic region of Uganda in which focus was on malaria and medicine use in nine primary schools, showed that in most cases, self-medication at home was the first response to malaria both in rural and urban areas in both teachers and students [21]. In the current and in previous studies [13,21], the main reasons for not seeking prescription included: the procedure is less costly, the respondents' similar experience with the same illness/drugs and being not satisfied with the services at the health facilities. It would be important for health officers to emphasize on seeking prescription and improve on the services at the health facilities in such malaria endemic regions.

Assessment of patterns of drug use revealed intriguing results. Although ACT and Quinine are the recommended anti-malarials in Kenya at the moment, the current data revealed that individuals with symptoms of malaria have easy access to medications which are non-efficacious to malaria, a phenomenon that had earlier been documented in other regions of the country [22,23]. For example, a study on the management of fevers in Kenyan children and adults in a region of seasonal transmission in Kenya found that majority (77.0%) of visitors to shops received a medicine with no anti-malarial efficacy at the time [23]. Surveys of treatment-seeking behaviours in three districts in Kenya of endemic malaria documented a similar access to drugs with minimal malarial efficacy being distributed by local shops for the treatment of individuals with symptoms of malaria [22]. As such, accessing anti-malarials which have lost efficacy in such malaria prone areas consisting of population of semi-immune adults raises a health concern, since the non-curative regimens may offer symptomatic relief without parasitological cure, a situation which would increase the malaria burden in the populations. Even when the recommended anti-malarial drugs are used, inappropriate treatment regimens, usually under-treatment, are common. In addition to non-compliance with correctly-prescribed regimens, much incorrect usage can be attributed to the practice of purchasing medications directly from informal sources without the benefit of any medical or prescribing advice, a practice that have been documented by other studies in Kenya [5,24]. The findings that under-treatment is frequent with doses of artemether/lumefantrine highlight the potential contribution of incorrect drug usage to the genesis of drug resistant malaria in these settings. As such, the government through their policies should initiate training of personnel at the sources (including those issuing drugs over the counters) on the importance of making available to the users, only the most effective antimalarial drugs with proper prescription. It is anticipated that this information will further trickle down to the end user and lead to correct usage of the anti-malarials.

## Conclusion

Even though combination therapy looks promising to fight drug resistant malaria, knowledge on the importance of adherence as well as availability of other less effective anti-malarials in the market may be obstacles to effective utilization of the therapy. This may adversely affect up-scaling of the WHO new treatment policy on malaria. It will be important for interventions to be directed at creating awareness about changes in recommended drugs so as to lessen the use of less efficacious anti-malarials. In addition, the consumers need to be educated on the importance of drug adherence in such areas to reduce the emergence and spread of drug-resistant malaria.

## Competing interests

The authors declare that they have no competing interests.

## Authors' contributions

CAW designed, carried out the survey studies in the peri-urban population and participated in the drafting of the manuscript. WGZOJ, ER and CO designed the study and participated in the drafting of the manuscript. DK and BA performed the statistical analyses. All authors read and approved the final manuscript.
